# The Western New York Dental Association

**Published:** 1867-11

**Authors:** 


### Proceedings of Societies.

### THE WESTERN NEW YORK DENTAL ASSOCIATION.

The Western New York Dental Association held its Fifth Annual Meeting in Sons of Temperance Hall, in Lockport, commencing on Tuesday, Oct. 1, 1867, and continuing two days. Although but thinly attended, it was a session of unusual interest to those present. Much of this was owing to the presence of several Dentists from abroad, among whom was Prof. Taft, of the Ohio College of Dental Surgery.

The retiring President, Dr. R. G. Snow, read the annual address, a copy of which was requested by the Association for publication in the DENTAL REGISTER.*

At the annual election of officers Dr. L. J. Walter, of Lockport, was elected President; Dr. A. P. Southwick, of Buffalo, Vice President; Dr. J. Requa, of Rochester, Treasurer; and Dr. W. C. Barrett, of Warsaw, Secretary. The subjects for discussion were:

1st—The manner of preparing teeth for filling, and the reasons for annealing gold. Essayist, Dr. A. P. Southwick.

2d—The different methods of preparing gold, and their relative merits. Essayist, Dr. G. C. Daboll.

3d—Mechanical Dentistry. Essayist, Dr. W. C. Barrett,

4th—Management, and the best means of preventing the destruction of the deciduous teeth. Essayist, Dr. Frank French.

5th—Materia Medica, and its relations to Dental practice. Essayist, Dr. L. W. Bristol.

Animated discussions were held upon all of these subjects, eliciting some original methods of practice.

---

* For which see the first article of this number.—ED.

Dr. Taft gave a history of the effort to induce the Ohio Legislature to pass a bill regulating the practice of Dentistry in that State. A committee was appointed to agitate the subject in the State of New York. Resolutions were introduced condemning the practice of gold beaters putting up their foil under other than their own stamp, and recommending those beaters who have uniformly refused to do so, to the patronage of the profession.

Dr. Hayes introduced a new device for the more perfect packing of rubber plates, and showed very perfect specimens of its work.

After a vote of thanks to Prof. Taft for his kindness in throwing up business engagements to attend the meeting of the Association, and to Dr. D. J. Walter, and L. W. Bristol, of Lockport, for their hospitality in entertaining the profession, the Association adjourned to meet at Buffalo, on the first Tuesday in May, 1868.

W. C. BARRETT, *Secretary.*

